# Transcriptional Regulation of *RIP2* Gene by NFIB Is Associated with Cellular Immune and Inflammatory Response to APEC Infection

**DOI:** 10.3390/ijms23073814

**Published:** 2022-03-30

**Authors:** Hongyan Sun, Naying Li, Jishuang Tan, Huan Li, Jibin Zhang, Lujiang Qu, Susan J. Lamont

**Affiliations:** 1 College of Animal Science and Technology, Yangzhou University, Yangzhou 225009, China; bio0407o07@163.com (N.L.); sqbreedingg@126.com (J.T.); 2 Joint International Research Laboratory of Agriculture & Agri-Product Safety, Ministry of Education, Yangzhou University, Yangzhou 225009, China; 3 School of Biological and Chemical Engineering, Yangzhou Polytechnic College, Yangzhou 225009, China; 4 Department of Pathology, City of Hope National Medical Center, Duarte, CA 91006, USA; jibinzhang12@gmail.com; 5 College of Animal Science and Technology, China Agricultural University, Beijing 100091, China; quluj@163.com; 6 Department of Animal Science, Iowa State University, Ames, IA 50011, USA; lamontsj@126.com

**Keywords:** APEC, *RIP2*, *NFIB*, gene expression, apoptosis, immune response

## Abstract

Avian pathogenic *E. coli* (APEC) can cause localized or systemic infection, resulting in large economic losses per year, and impact health of humans. Previous studies showed that *RIP2* (receptor interacting serine/threonine kinase 2) and its signaling pathway played an important role in immune response against APEC infection. In this study, chicken HD11 cells were used as an in vitro model to investigate the function of chicken *RIP2* and the transcription factor binding to the *RIP2* core promoter region via gene overexpression, RNA interference, RT-qPCR, Western blotting, dual luciferase reporter assay, CHIP-PCR, CCK-8, and flow cytometry assay following APEC stimulation. Results showed that APEC stimulation promoted *RIP2* expression and cells apoptosis, and inhibited cells viability. Knockdown of *RIP2* significantly improved cell viability and suppressed the apoptosis of APEC-stimulated cells. Transcription factor NFIB (Nuclear factor I B) and GATA1 (globin transcription factor 1) binding site was identified in the core promoter region of RIP2 from −2300 bp to −1839 bp. However, only NFIB was confirmed to be bound to the core promoter of *RIP2*. Overexpression of *NFIB* exacerbated cell injuries with significant reduction in cell viability and increased cell apoptosis and inflammatory cytokines levels, whereas opposite results were observed in *NFIB* inhibition treatment group. Moreover, *RIP2* was up-regulated by *NFIB* overexpression, and *RIP2* silence mitigated the effect of *NFIB* overexpression in cell apoptosis, inflammation, and activation of NFκB signaling pathways. This study demonstrated that *NFIB* overexpression accelerated APEC-induced apoptosis and inflammation via up-regulation of *RIP2* mediated downstream pathways in chicken HD11 cells.

## 1. Introduction

Avian pathogenic *E. coli* (APEC), a subset of extraintestinal pathogenic *E. coli* (ExPEC), is the causative agent of several localized or systemic syndromes, affecting poultry of any age and production categories. The most common infections caused by APEC are acute septicemia in young birds or diverse diseases such as subacute pericarditis, airsacculitis, salpingitis, peritonitis, and cellulitis for young survivors, leading to high morbidity, mortality, and carcass contamination in broiler, layer, game, and turkey [1,2,3]. APEC causes economic losses of millions of dollars worldwide. For example, as one of the world’s largest exporters of chicken meat, Brazil has 45.2% of poultry meat export rejected due to APEC infections [4].

Moreover, previous studies have demonstrated that uropathogenic *E. coli* (UPEC) causing human urinary tract infections shared genetic similarities with APEC [5,6,7], indicating APEC is potentially zoonotic and can cause various health hazards. It has been reported that foodborne pathogens have led to an estimated 4 million cases of human illness in Canada [8]. APEC is one of the foodborne zoonotic pathogens most frequently associated with infections from poultry products [9,10]. Therefore, it is of great interest to improve our understanding of host immune response to APEC and to formulate effective preventive and treatment strategies. Although antibiotics can be used to control diseases caused by APEC, most current APEC strains are resistant to different classes of antibiotics. For example, more than 80% of APEC strains in China, and 92% in Europe, United States, and Australia, are resistant to antibiotics [11,12]. Furthermore, effective vaccines are still not available to protect chickens against APEC infections mainly due to the diversity of APEC serotypes [13]. Therefore, genetics improvement of chickens is a potentially effective and sustainable way in the fight against APEC.

Currently, a large number of studies have been performed on poultry response to APEC infection at the genomic and transcriptomic levels. Previous transcriptomic investigations suggest that the NOD-like receptor signaling pathway and its key regulator *RIP2* (receptor interacting serine/threonine kinase 2) are significantly activated in bone marrow, thymus, bursa, leukocytes in blood, and spleen in chickens with APEC infection [14,15,16,17,18]. The *RIP2* gene, also known as *RIPK2* or *RICK*, plays an important role in mucosal immunity in the respiratory system. Previous studies have found that *RIP2* was involved in mediating bacterial stimuli. For example, knockout of *RIP2* would result in a strong neutrophil recruitment to the lungs of *Legionella pneumophila* infected mice, and the expression levels of *RIP2* are critical to the production of cytokines [19]. Moreover, *RIP2* was found to be involved in the inflammation modulation and the clearance of *Listeria monocytogenes* [20], *Salmonella enterica* [21], and *Chlamydia pneumoniae* [22]. Although the aforementioned studies proved that *RIP2* has essential function in the process of immunity and bacterial clearance in mice and humans, the specific molecular mechanism of *RIP2* function and the factors regulating *RIP2* expression activity are still unknown.

Transcription factors, also known as trans-acting factors, are proteins with special structures that can bind to the specific DNA sequence (cis-acting element) in the upstream promoter region of the target gene to regulate gene expression in different tissues, cells, or environmental conditions at the transcriptional level to further modulate growth, development, and immunity [23,24,25]. For example, NFIB, a member of the nuclear factor 1 (NFI) family of transcription factors, is not only essential to activate viral genes, but also important to control expression of a large number of cellular genes [26]. It has been demonstrated that transcription factor NFIB can regulate proliferation and epithelial differentiation during lung maturation. The experiment of Hsu et al. [27] showed that knockout of *NFIB* would result in severe lung hypoplasia and developmental defects in the brain of mice. Moreover, *NFIB*, as a master regulator, can also coordinate epithelial-melanocyte stem cell proliferation and differentiation in hair follicles, and regulate the differentiation of neural progenitor cells in the brain [28,29]. Currently, several reports have suggested NFIB as an oncogene related to triple-negative breast cancer, squamous cell carcinoma of the esophagus, and submandibular gland carcinoma [30,31,32]. Another interesting transcription factor is GATA1 (globin transcription factor 1), which is critical to the regulation of cell growth and differentiation, cell survival, and maintenance of body functions [33,34]. Currently, it is still unclear whether NFIB and GATA1 can regulate the immune and inflammatory responses via a specific mechanism upon APEC infection. Additionally, it remains unknown whether the RIP2 can interact with NFIB or GATA1.

Therefore, in the present study, we aim to explore activity of chicken *RIP2* regulated by NFIB or GATA1 via analysis of its transcriptional regulatory mechanism, providing the potential therapeutic targets for controlling excessive inflammatory response upon APEC infection.

## 2. Results

### 2.1. Identification of NOD-like Receptor Signaling Pathway and RIP2 as APEC Regulator

**Figure 1:**
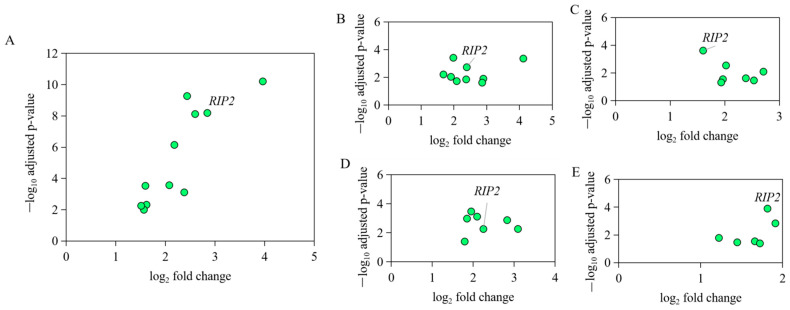
The expression level of genes in NOD-like receptor signaling pathway in different immune tissues of the same individual bird with APEC infection. (**A**) The expression level of *IL8L2*, *IL1B*, *IL18*, *RIP2*, *PSTPIP1*, *NOD1*, *HSP90B1*, *CARD9*, *MAPK1*, *MAPK11*, and *CASP8* in NOD-like receptor signaling pathway in bone marrow upon APEC infection based on the data of GSE67302. (**B**) The expression level of *IL8L2*, *IL18*, *IL8L1*, *MAPK12*, *MAPK11*, *BIRC2*, *CARD9*, *NOD1*, and *RIP2* in NOD-like receptor signaling pathway in thymus upon APEC infection based on the data of GSE69014. (**C**) The expression level of *MAPK8*, *IL18*, *TRAF6*, *HSP90AB1*, *NOD1*, *RIP2*, and *ITA* in NOD-like receptor signaling pathway in bursa upon APEC infection based on the data of GSE70334. (**D**) The expression level of *MAPK11*, *MAPK1*, *CASP8*, *ERBB2IP*, *IL8L2*, *RIP2*, and *NOD1* in NOD-like receptor signaling pathway in leukocytes in blood upon APEC infection based on the data of GSE31387. (**E**) The expression level of *IL1B*, *IL18*, *IL6*, *RIP2*, *NOD1*, and *CASP8* in NOD-like receptor signaling pathway in spleen upon APEC infection based on the data of GSE25511.

Based on the expression profile GSE67302, GSE6901, GSE70334, GSE31387, and GSE25511 of chicken bone marrow, thymus, bursa, leukocytes in blood, and spleen, respectively, from the same individuals and the same experiment (Table S1), the significant activation change of signaling pathway NOD-like receptor (NLR) was identified to be the most common transcriptomic response to APEC infection (Figure S1). As the key and core member of NOD-like receptor signaling pathway, *RIP2* expression level showed 4 to 10-fold changes upregulation in different tissues upon APEC infection (Figure 1), suggesting the important role of chicken *RIP2* in the immune response to APEC. This led us to explore the function and transcriptional regulation of chicken RIP2 after APEC infection.

### 2.2. APEC Increased RIP2 Expression and Suppressed Chicken HD11 Cells Viability

**Figure 2:**
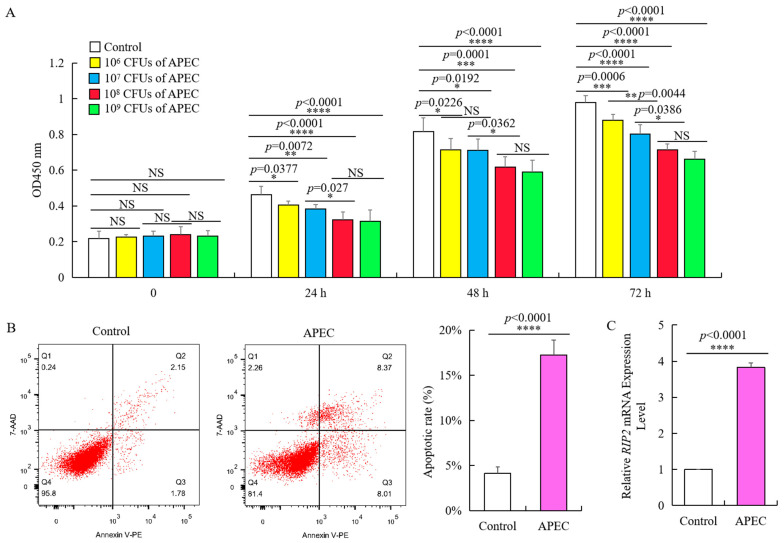
APEC promoted *RIP2* expression and apoptosis, and suppressed the viability of chicken HD11 cells. (**A**) Viability of chicken HD11 cells analyzed at 24, 48, and 72 h after APEC infection (data are shown as mean ± SD, *n* = 5 independent experiments. * *p* < 0.05, ** *p* < 0.01, *** *p* < 0.001, and **** *p* < 0.0001, one-way ANOVA, LSD. OD, optical density). (**B**) Flow cytometry was used to detect apoptosis of chicken HD11 cells treated with APEC at 24 h (data are shown as mean ± SD, *n* = 5 independent experiments. **** *p* < 0.0001, one-way ANOVA, LSD). (**C**) *RIP2* expression in APEC-treated chicken HD11 cells after 24 h was determined using RT-qPCR (data are shown as mean ± SD, *n* = 5 independent experiments. **** *p* < 0.0001, one-way ANOVA, LSD).

Chicken HD11 cells were cultured with APEC to examine cellular immune response. This is further supported by the flow cytometry through apoptotic cell makers staining of chicken HD11 cells treated for 24 h with PE Annexin V and 7-aminoactinomycin D (7-AAD), which shows significant increase in apoptotic rate from around 4 to 17% (Figure 2B). Moreover, treatment with APEC substantially promoted an almost four-fold increase We performed the HD11 cells infection with different dose of APEC colony forming units (CFUs) experiment for 0 h, 24 h, 48 h, and 72 h, and then examined cell viability. As shown by the viability assay with OD 450 nm, 1 × 10^6^ CFUs ~ 1 × 10^9^ CFUs of APEC can significantly impair cell viability with a dose-dependent manner at different time points despite the time-dependent growth of the HD11 cell after stimulation (Figure 2A). There was significant reduction in cell viability when the APEC concentration increased from 1 × 10^7^ CFUs to 1 × 10^8^ CFUs at 24 h, 48 h, and 72 h, while no significance existed between 1 × 10^8^ CFUs of APEC and 1 × 10^9^ CFUs of APEC (Figure 2A). Thus, 0.1 mL 1 × 10^8^ CFUs of APEC can significantly suppress the proliferation of chicken HD11 cells and promote cell apoptosis (Figure 2A,B).This is further supported by the flow cytometry through apoptotic cell makers staining of chicken HD11 cells treated for 24 h with PE Annexin V and 7-aminoactinomycin D (7-AAD), which shows significant increase of apoptotic rate from around 4 to 17% (Figure 2B). Moreover, treatment with APEC substantially promoted an almost four-fold increase in *RIP2* expression (from 0.98 to 3.87, Figure 2C).

### 2.3. RIP2 Knockdown Rescued the APEC-Stimulated Effect on Viability and Apoptosis of Chicken HD11 Cells

**Figure 3:**
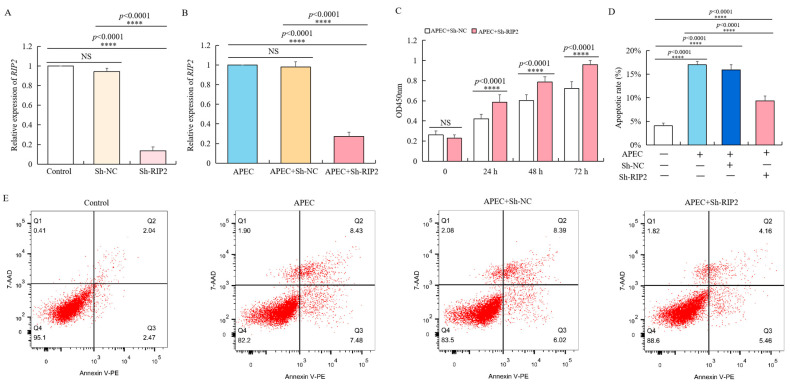
*RIP2* knockdown reverses the APEC-induced effects on viability and apoptosis of chicken HD11 cells. (**A**) RT-qPCR analysis of *RIP2* expression in normal HD11 cells, and HD11 cell transfected with negative control (Sh-NC), and Sh-RIP2 (data are shown as mean ± SD, *n* = 5 independent experiments. **** *p* < 0.0001, one-way ANOVA, LSD). (**B**) RT-qPCR analysis of *RIP2* expression in normal HD11 cells, and HD11 cell transfected with negative control (Sh-NC), and Sh-RIP2 chicken HD11 cell at 24 h post APEC treatment (data are shown as mean ± SD, *n* = 5 independent experiments. **** *p* < 0.0001, one-way ANOVA, LSD). (**C**) Viability was evaluated in chicken HD11 cells transfected with either Sh-NC or Sh-RIP2 at 24, 48, and 72 h after treatment with APEC (data are shown as mean ± SD, *n* = 5 independent experiments. **** *p* < 0.0001, one-way ANOVA, LSD). (**D**) Apoptotic rate of chicken HD11 cells treated with APEC and transfected with Sh-NC or Sh-RIP2 (data are shown as mean ± SD, *n* = 5 independent experiments. **** *p* < 0.0001, one-way ANOVA, LSD). (**E**) Flow cytometry was used to detect the apoptosis change of chicken HD11 cells after treatment with APEC and transfection with Sh-NC or Sh-RIP2.

To verify the regulatory effect of *RIP2* in cell immune response, we used lentivirus vector to transfect chicken HD11 cells with Sh-RIP2, of which the knockdown efficiency was about 90% of the background compared to negative control Sh-NC (Figure 3A). Even with APEC stimulation for 24 h, the expression of *RIP2* in Sh-RIP2 transfected chicken HD11 cells was significantly lower (Figure 3B). We observed that *RIP2* knockdown promoted the growth of APEC-treated chicken HD11 cells. Specifically, the optical density (OD 450 nm) values of Sh-RIP2 were consistently higher than that of Sh-NC at 24, 48, and 72 h after APEC stimulation (Figure 3C). Moreover, flow cytometry showed that *RIP2* knockdown inhibited the apoptosis of APEC-stimulated chicken HD11 cells (Figure 3D,E). The apoptotic ratios dropped from 16% to about 9% after *RIP2* knockdown. Therefore, *RIP2* knockdown rescued detrimental effects caused by APEC treatment.

### 2.4. Identification of the Chicken RIP2 Promoter Region and Analysis of Transcriptional Regulatory Elements

To investigate the core promoter region of *RIP2*, the chicken DF1 and HD11 cell lines were transfected with recombinant plasmids and the internal control pRL-TK plasmid. The pGL3-basic plasmid was used as a negative control. The activity of chicken *RIP2* promoter was identified using the dual-luciferase assay. Cloning of the chicken *RIP2* gene promoter was started from 3000 bp upstream to 200 bp downstream of transcriptional start site.

**Figure 4:**
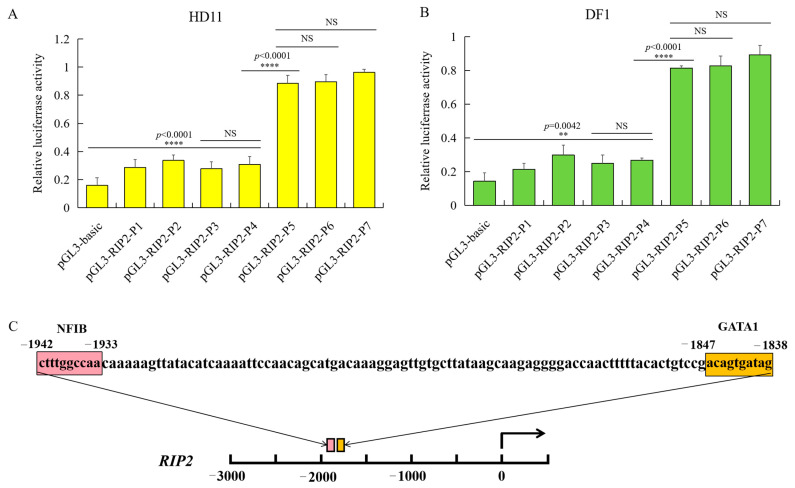
Identification of chicken *RIP2* promoter through luciferase activity of the different inserted fragments and prediction of regulatory elements. (**A**) Luciferase activity of the different inserted fragments of the chicken *RIP2* promoter in HD11 cells (data are shown as mean ± SD, *n* = 5 independent experiments. **** *p* < 0.0001, one-way ANOVA, LSD). (**B**) Luciferase activity of the different inserted fragment of the chicken *RIP2* promoter in DF1 cells (data are shown as mean ± SD, *n* = 5 independent experiments. ** *p* < 0.01, **** *p* < 0.0001, one-way ANOVA, LSD). (**C**) Prediction of binding sites for transcription factors at −1839 to +54 bp of *RIP2* in the genomic assembly of *Gllus gallus* 6.0.

The promoter activity of chicken *RIP2* showed the same trend in both DF1 and HD11 cell lines. The recombinant plasmid pGL3-P5 (−2300/+38), pGL3-P6 (−2750/+38), and pGL3-P7 (−3162/+38) had significantly higher promoter activity compared to the pGL3-basic, pGL3-P1 (−439/+38), pGL3-P2 (−962/+38), pGL3-P3 (−1389/+38), and pGL3-P4 (−1839/+38). There was no significant difference between each of the two groups in pGL3-P5, pGL3-P6, and pGL3-P7. The promoter activity of pGL3-P5 was sharply increased compared to pGL3-P4 (Figure 4A), indicating that the core promoter region was between −2300 bp and −1839 bp. The bioinformatics online software PROMO [35] (http://alggen.lsi.upc.es/cgi-bin/promo_v3/promo/promoinit.cgi?dirDB=TF_8.3, 20 August 2021) and AliBaba [36] (http://gene-regulation.com/pub/programs/alibaba2/index.html, 20 August 2021) were used to identify putative activator or repressor regulatory elements in the core promoter region. As a result, two essential transcription factors—NFIB and GATA1—were predicted to have binding sites at −2300 bp ~ −1839 bp (Figure 4B).

### 2.5. Identification of Transcription Factors in Core Region of Chicken RIP2 Promoter

**Figure 5:**
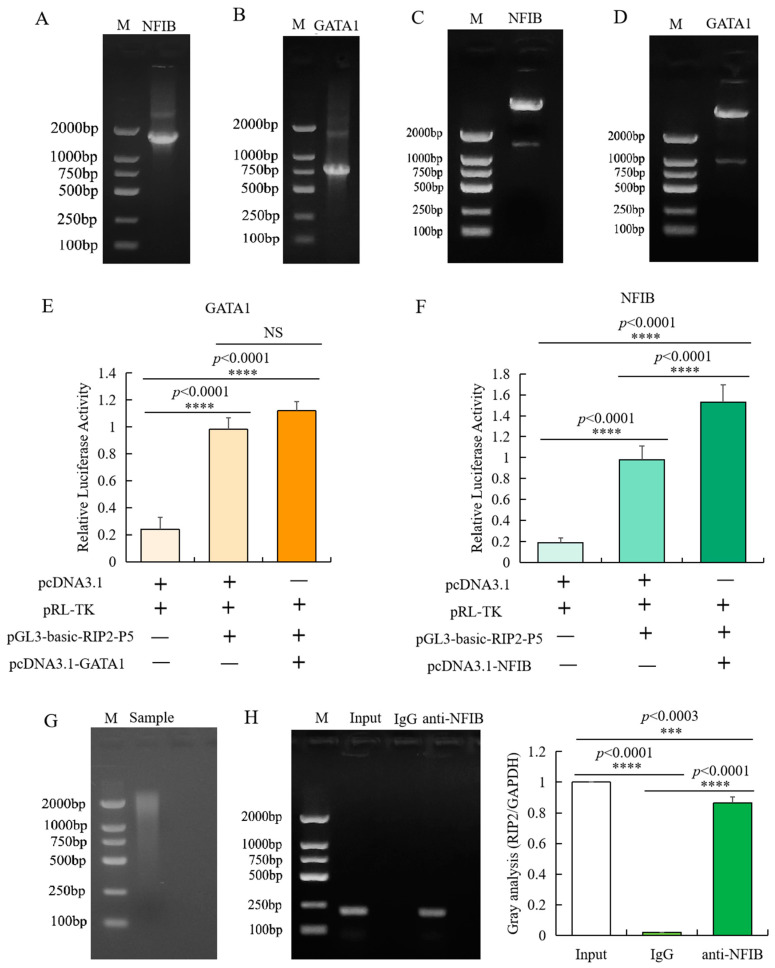
Identification of transcription factor binding sites in the promoter of *RIP2*. (**A**,**B**) Agarose gel electrophoresis of amplified NFIB (**A**) and GATA1 (**B**) insertion. (**C**,**D**) Identification of inserted fragment in pcDNA3.1-GATA1 (**C**) and pcDNA3.1-NFIB (**D**) through double enzyme digestion. (**E**) Luciferase activity analysis of *RIP2* promoter in different conditions with or without pcDNA3.1-GATA1 (data are shown as mean ± SD, *n* = 5 independent experiments. **** *p* < 0.0001, one-way ANOVA, LSD). (**F**) Luciferase activity analysis of *RIP2* promoter in different conditions with or without pcDNA3.1-NFIB (data are shown as mean ± SD, *n* = 5 independent experiments. **** *p* < 0.0001, one-way ANOVA, LSD). (**G**) Agarose gel electrophoresis analysis of the sonicated DNA fragments for CHIP-PCR experiment. (**H**) Binding of NFIB to the promoter region of RIP2 in chicken HD11 cells analyzed by ChIP-PCR (Input is the PCR amplification product of the sample without immunoprecipitation reaction (input control); IgG is the PCR amplification product of mouse IgG antibody (negative control); anti-NFIB is the PCR amplification product of fragments bound by NFIB and isolated with NFIB antibody; data are shown as mean ± SD, *n* = 3 independent experiments. *** *p* < 0.001, **** *p* < 0.0001, one-way ANOVA, LSD).

To confirm that the transcription factor NFIB and GATA1 binds to the different promoter region of *RIP2* gene, two overexpression recombinant plasmids of the transcription factor NFIB (pcDNA3.1-NFIB) and GATA1 (pcDNA3.1-GATA1) were constructed, respectively. Results of agarose gel electrophoresis of amplified insertion (Figure 5A,B), double enzyme digestion of recombinant plasmids (Figure 5C,D), and sequencing showed that the two recombinant plasmids (pcDNA3.1-NFIB and pcDNA3.1-GATA1) were successfully constructed. Then, pcDNA3.1-NFIB or pcDNA3.1-GATA1, together with RIP2 promoter recombinant plasmid pGL3-P5, were used to transfect chicken HD11 cells. Results showed that the overexpression plasmid pcDNA3.1-NFIB can significantly improve the activity of the *RIP2* promoter (Figure 5F), while the pcDNA3.1-GATA1 plasmids had no significant effect on the activity of chicken *RIP2* promoter (Figure 5E). It can be speculated that the transcriptional factor NFIB may bind to the promoter region of chicken *RIP2* gene to enhance promoter activity. Then, we further investigated the binding status of NFIB and *RIP2* genes in vivo.

CHIP-PCR was used to identify the specific binding of transcription factor NFIB to the core promoter region of chicken *RIP2* gene in vivo. Based on the region of the core promoters of chicken *RIP2* that contains the putative NFIB binding sites, PCR primers were designed with an amplification size of 196 bp and the immunoprecipitated DNA fragments were used as template. Target amplification was not detected in the negative control, but was detected in the input control, indicating that the experimental results were reliable and correct. The target fragment was obtained by PCR amplification of the DNA fragments immunoprecipitated by NFIB antibody (Figure 5G,H). Meanwhile, the target amplified product was sequenced and confirmed to be correct. These data confirmed that the transcription factor NFIB bound specifically to the core regions of the promoters of *RIP2* gene in chicken HD11 macrophages.

### 2.6. Design and Screening of siRNA Interfering with NFIB

**Figure 6:**
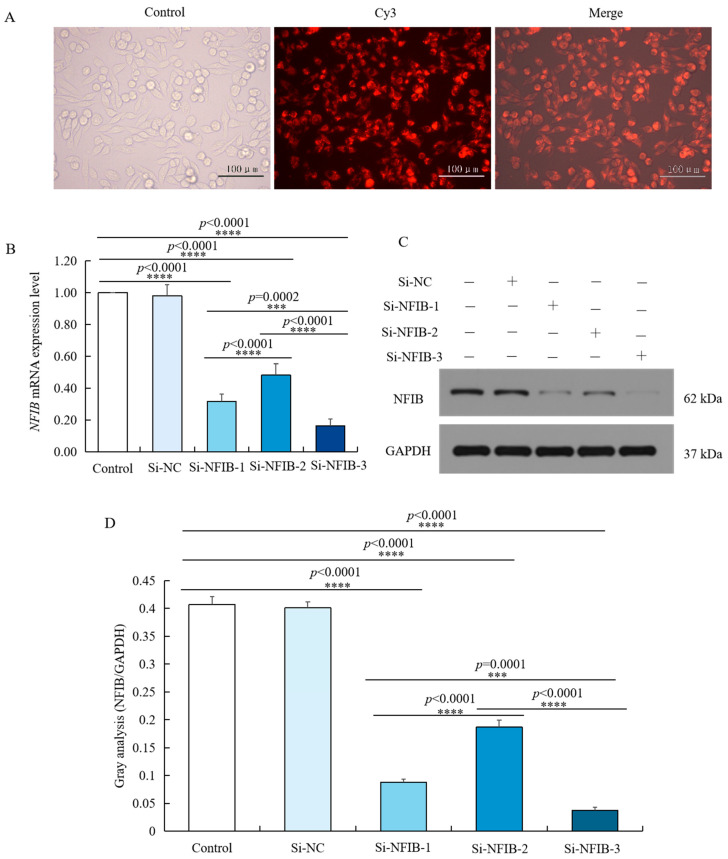
siRNA knockdown of *NFIB* expression in chicken HD11 cells. (**A**) Transfection efficiency was tested by small interfering RNA under a fluorescence microscope. (**B**) Levels of *NFIB* mRNA expression in chicken HD11 cells transfected with Si-NFIB-1, Si-NFIB-2, or Si-NFIB-3, and the control groups (data are shown as the mean ± SD, *n* = 5 independent experiments. *** *p* < 0.001, **** *p* < 0.0001, one-way ANOVA, LSD). (**C**) Western blotting of protein extracted from chicken HD11 cells transfected with Si-NFIB-1, Si-NFIB-2, or Si-NFIB-3 and the control groups; GAPDH was included as a loading control. (**D**) Image J software was used for gray-level analysis of NFIB in HD11 cells transfected with Si-NFIB-1, Si-NFIB-2, or Si-NFIB-3 and the control groups (data are shown as the mean ± SD, *n* = 3 independent experiments. *** *p* < 0.001, **** *p* < 0.0001, one-way ANOVA, LSD).

According to the sequence of *NFIB*, three pairs of double-stranded siRNAs were designed, which were located at the position of 1119 bp (Si-NFIB-1), 1612 bp (Si-NFIB-2), and 1259 bp (Si-NFIB-3). Si-NFIB-1, Si-NFIB-2, Si-NFIB-3, and Si-NC were, respectively, transfected into chicken HD11 cells. After 48 h, the transfection efficiency was over 95% as displayed in Figure 6A. Total RNA of chicken HD11 cells was extracted from different treatment groups, and the mRNA expression of *NFIB* was measured by using *GAPDH* as an internal reference as shown in Figure 6B. After transfection with Si-NFIB-1, Si-NFIB-2, and Si-NFIB-3 in chicken HD11 cells, the mRNA expression of *NFIB* was significantly decreased (*p* < 0.0001). Among the three siRNA constructs, Si-NFIB-3 had the best interference effect. Results of Western blot showed similar trends as mRNA expression (Figure 6C,D). Thus, Si-NFIB3 was used to knock down NFIB in chicken HD11 cells to investigate the effect of this gene on *RIP2* gene and cellular immune inflammatory response in the following experiment.

### 2.7. RIP2 Was Positively Regulated by NFIB Expression upon APEC Infection

**Figure 7:**
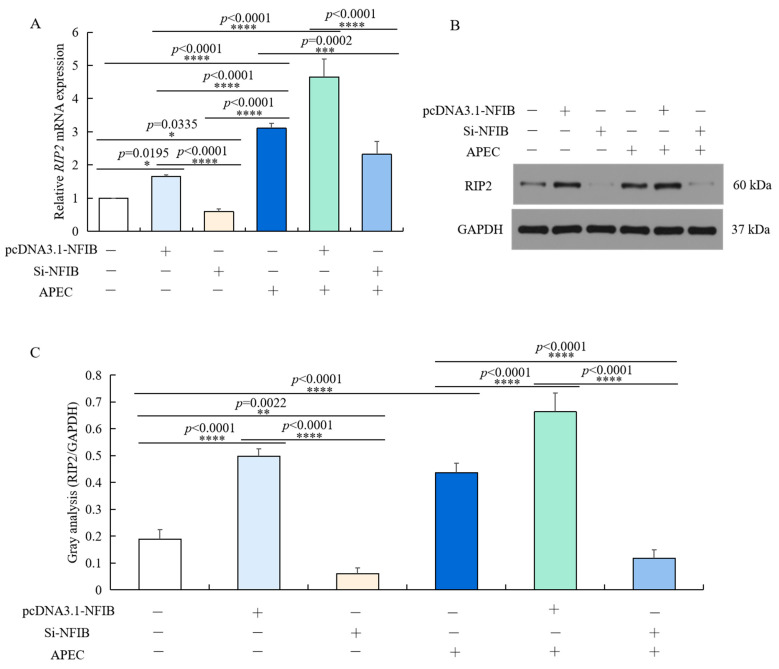
*RIP2* expression level was positively modulated by *NFIB* expression in HD11 cells regardless of APEC infection. (**A**) The mRNA expression of *RIP2* was detected in HD11 cells with overexpression or knockdown of *NFIB* in comparison to negative controls under conditions with or without APEC treatment by RT-qPCR (data are shown as the mean ± SD, *n* = 5 independent experiments. * *p* < 0.05, *** *p* < 0.001, and **** *p* < 0.0001, one-way ANOVA, LSD). (**B**) The protein expression of RIP2 in the same experiment was measured by Western blot. (**C**) Image J software was used for gray-level analysis of RIP2 in different treatments in the same experiment (data are shown as the mean ± SD, *n* = 3 independent experiments. ** *p* < 0.01, **** *p* < 0.0001, one-way ANOVA, LSD).

Chicken HD11 cells were transfected using Si-NFIB or pcDNA3.1-NFIB for 48 h and incubated with APEC for 24 h. As shown in Figure 7A, although the mRNA expression of *RIP2* was significantly upregulated by APEC infection. In both HD11 treated with or without APEC, *RIP2* expression was markedly down-regulated in chicken HD11 cells transfected with Si-NFIB (*p* = 0.0002 or *p* = 0.0335) and further upregulated in chicken HD11 cells transfected with pcDNA3.1-NFIB (*p* < 0.0001, or *p* = 0.0195), in comparison to normal control. The protein expression changes were similar to the mRNA expression, as *NFIB* silence decreased the expression of *RIP2* and *NFIB* overexpression elevated *RIP2* levels no matter whether the cells were infected with APEC (Figure 7B). These results showed that *RIP2* expression was positively regulated by *NFIB* in chicken HD11 cells.

### 2.8. NFIB Exacerbated APEC-Induced Injuries through Modulation of RIP2 in Chicken HD11 Cells

**Figure 8:**
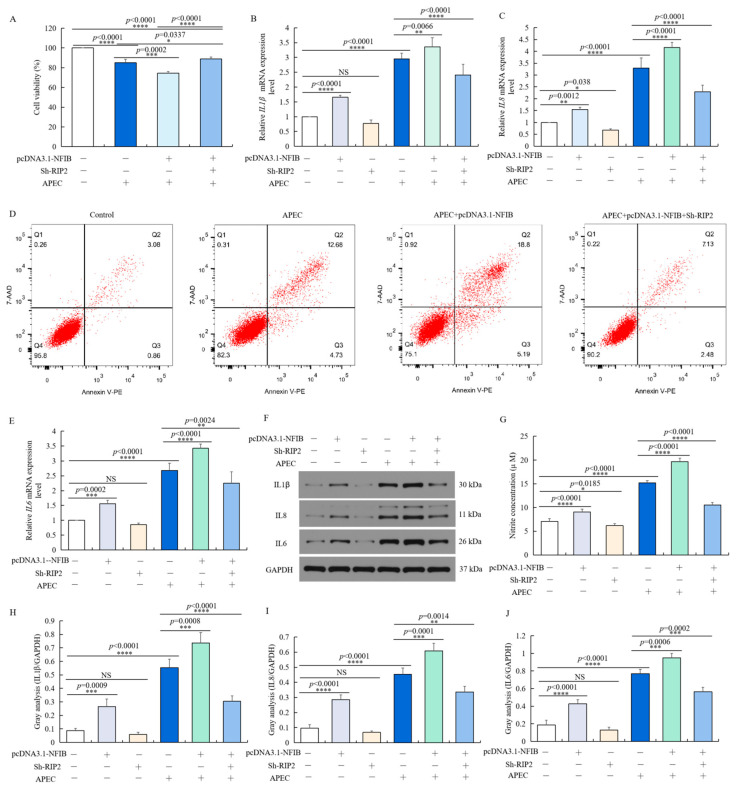
*NFIB* enhanced APEC-induced injuries through modulation of *RIP2* in chicken HD11 cells. Chicken HD11 cells were transfected with Sh-RIP2, pcDNA3.1-NFIB, APEC, co-transfected with pcDNA3.1-NFIB and APEC, or co-transfected with pcDNA3.1-NFIB, Sh-RIP2, and APEC, respectively. (**A**) Cell viability was measured by cell counting kit-8 (CCK8) in different treatment conditions (data are shown as the mean ± SD, *n* = 5 independent experiments. * *p* < 0.05, *** *p* < 0.001, and **** *p* < 0.0001, one-way ANOVA, LSD). (**B**) The mRNA expression level of *IL1β* in different treatment conditions (data are shown as the mean ± SD, *n* = 5 independent experiments. ** *p* < 0.01, **** *p* < 0.0001, one-way ANOVA, LSD). (**C**) The mRNA expression level of *IL8* in different treatment conditions (data are shown as the mean ± SD, *n* = 5 independent experiments. * *p* < 0.05, ** *p* < 0.01, and **** *p* < 0.0001, one-way ANOVA, LSD). (**D**) Cell apoptosis was measured by flow cytometry with annexin V-PE/7-AAD in different treatment conditions. (**E**) The mRNA expression level of *IL6* in different treatment conditions (data are shown as the mean ± SD, *n* = 5 independent experiments. ** *p* < 0.01, *** *p* < 0.001, and **** *p* < 0.0001, one-way ANOVA, LSD). (**F**) The protein expression of IL1β, IL8, and IL6 was measured by Western blotting with different treatment conditions. (**G**) Nitric oxide production in different treatments (data are shown as the mean ± SD, *n* = 5 independent experiments. * *p* < 0.05, **** *p* < 0.0001, one-way ANOVA, LSD). (**H**–**J**) Image J software was used for gray-level analysis of protein expression of IL1β (**H**), IL8 (**I**), and IL6 (**J**) in different treatments (data are shown as the mean ± SD, *n* = 3 independent experiments. ** *p* < 0.01, *** *p* < 0.001, and **** *p* < 0.0001, one-way ANOVA, LSD).

We then explored whether *NFIB* plays a role in APEC-injured chicken HD11 cells via regulating *RIP2* expression. It was found that the effect of *NFIB* overexpression upon APEC-induced injuries was reversed by *RIP2* knockdown in chicken HD11 cells. *NFIB* overexpression significantly reduced cell viability (*p* < 0.0001 or *p* = 0.0002) (Figure 8A), increased apoptotic cell rates (Figure 8D), enhanced the NO production (*p* < 0.0001 or *p* < 0.0001) (Figure 8G), and promoted the mRNA and protein expression levels of IL1β (*p* < 0.0001 or *p* = 0.0066) (Figure 8B,H), IL8 (*p* < 0.0001 or *p* < 0.0001) (Figure 8C,I) and IL6 (*p* < 0.0001 or *p* < 0.0001) (Figure 8E,J) in APEC-injured chicken HD11 cells (Figure 8F), in comparison to respective control with or without APEC treatment. *RIP2* silence with Sh-RIP2 after transfection with pcDNA3.1-NFIB exhibited an antagonistic effect with increased cell viability (*p* < 0.0001), reduced cell apoptosis and NO production (*p* < 0.0001), as well as the mRNA and protein expression levels of proinflammatory factor IL1β (*p* < 0.0001), IL8 (*p* = 0.0014), and IL6 (*p* = 0.0002) in APEC-injured chicken HD11 cells (Figure 8). Generally, these results revealed that *NFIB* worsened APEC-induced injuries through modulation of *RIP2* in chicken HD11 cells, and these injuries could be mitigated by knockdown of *RIP2*.

### 2.9. NFIB Overexpression Activated NFκB via Up-Regulating RIP2

**Figure 9:**
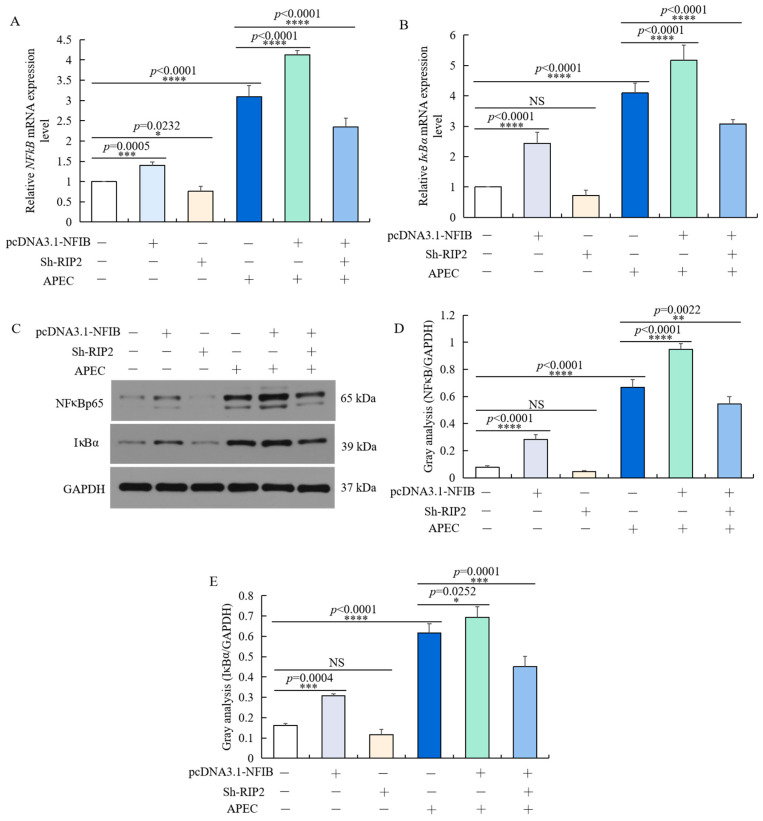
*NFIB* overexpression activated NFκB signaling pathway by up-regulating *RIP2*. Chicken HD11 cells were transfected with Sh-RIP2, pcDNA3.1-NFIB, and APEC, co-transfected with pcDNA3.1-NFIB and APEC, or co-transfected with pcDNA3.1-NFIB, Sh-RIP2, and APEC. (**A**,**B**) The mRNA expression of *NFκB*p65 (**A**) and *IκBα* (**B**) was detected by RT-qPCR (data are shown as the mean ± SD, *n* = 5 independent experiments. * *p* < 0.05, *** *p* < 0.001, and **** *p* < 0.0001, one-way ANOVA, LSD). (**C**) The protein expression of NFκBp65 and IκBα were measured by Western blot. (**D**,**E**) Image J software was used for gray-level analysis of NFκBp65 (**D**) and IκBα (**E**) in different treatments (data are shown as the mean ± SD, *n* = 3 independent experiments. * *p* < 0.05, ** *p* < 0.01, *** *p* < 0.001 and **** *p* < 0.0001, one-way ANOVA, LSD).

As we found that *NFIB* can regulate APEC-induced chicken HD11 cell injuries through regulation of *RIP2* activity, we hypothesized that *NFIB* might regulate the *RIP2* downstream signaling pathway—NFκB and IκB—to further regulate the cellular immune and inflammatory response. As shown in Figure 9, inhibition of *RIP2* reduced the mRNA and protein expression level of NFκB p65 (Figure 9A,C,D) and IκBα (Figure 9B–D), which weakened the effect of *NFIB* overexpression on NFκB pathway. The two factors showed similar change as RIP2, IL1β, IL8, and IL6 in different treatment conditions. In conclusion, these results indicated that *NFIB* overexpression activated NFκB by upregulation of *RIP2* in APEC-injured chicken HD11 cells.

## 3. Discussion

The immune response of poultry to avian pathogenic *E. coli* (APEC) is a complex process regulated by many factors. Our previous research has indicated that the *RIP2* and its signaling pathway had the ability to regulate chicken immune response to APEC infection [14,15,16,17,18]. Herein, APEC-stimulation was performed in vitro, which can induce chicken HD11 macrophages apoptosis and increase *RIP2* expression level. Then, we further confirmed knockdown of *RIP2* can alleviate the APEC-induced injury of chicken HD11 macrophages, which is consistent with those findings in humans and mice [37,38]. Previous studies have reported that knockdown of *RIP2* could inhibit NFκB signaling, reduce levels of anti-apoptotic proteins, and sensitize cells to drug treatment of triple negative breast cancer [39]. Bist et al. also demonstrated that inhibiting *RIP2* gene can reduce the release of various inflammatory factors and alleviate inflammatory response syndrome upon acinetobacter baumannii infection [40]. Although the function of *RIP2* has been known for decades in humans and mice, the factors regulating *RIP2* expression activity have not been studied.

Gene expression is a complex process regulated by multiple factors. Whether a gene is expressed or not often depends on the specific promoter initiation process [41]. A promoter is the DNA sequence involved in the transcription of a specific gene and its regulation, which can be recognized by RNA polymerase and initiate gene transcription with the assistance of transcription factors [42]. To explore the factors regulating *RIP2* expression activity, we firstly analyzed and identified the core promoter region of chicken *RIP2*. Luciferase reporter gene vector utilized in this study is a common method for many studies of the promoter activity and core regulatory regions [43,44,45]. Meanwhile, chicken HD11 and DF1 cells were used as experimental models to double check the promoter activity of *RIP2*. Chicken HD11 macrophage is an immortalized cell line formed by transforming chicken bone marrow cells through replication deficient avian leukemia virus MC29 strain [46], which is commonly used in the research of animal husbandry and veterinary medicine. DF1 is a continuous cell line of chicken embryo fibroblasts. The cells are free of endogenous sequences related to avian sarcoma and leukosis viruses and have normal fibroblastic morphology [47]. As similar results were obtained from chicken HD11 and DF1 cells, it is reasonable to believe that the core promoter region of chicken *RIP2* is −2300/−1839 bp.

A promoter itself does not control gene activity; it regulates gene activity through the binding of transcription factor binding site in promoter to transcription factor [48,49]. Herein, bioinformatics analysis predicted that the core promoter region (−2300/−1839 bp) of *RIP2* gene contains binding sites for crucial transcription factor NFIB and GATA1. Our further experiments demonstrated that NFIB exhibited the strongest binding to the promoter region of *RIP2* in vivo and in vitro to positively regulate the *RIP2* activity. Recently, NFIB was identified as an oncogenic molecule in several cancers, which is highly expressed in tumor cells and modulates cellular proliferation, migration, invasion, and apoptosis. Several researchers have demonstrated that as a versatile regulator of cell differentiation, *NFIB* up-regulated its expression in gastric cancer [50], small-cell lung cancer [51], melanoma [52], and breast cancer [53], whereas paradoxically *NFIB* exhibits tumor suppressive functions in cutaneous squamous cell carcinoma and glioblastoma [54,55]. In the current study, we firstly identified that *NFIB* was involved in chicken HD11 cells apoptosis, the expression of inflammatory cytokines, and NO production upon APEC infection. Although there is no previous studies directly proving that *NFIB* is directly related to bacterial infection, the results obtained right now were reasonable and explainable. As we know, APEC can cause severe respiratory and systemic disease in chicken. *NFIB* is essential for both lung maturation and brain development [56,57,58]. More importantly, recent evidence indicates that *NFIB* was involved in the inflammatory response and the apoptosis of cardiomyocytes under the regulation of miR-346 [59]. The aforementioned studies are consistent with current results. Our study suggested that *NFIB* exerted a pivotal role in chicken HD11 cells’ immune and inflammatory response upon APEC infection. However, by what mechanism does *NFIB* regulate cellular immune and inflammatory response?

Wu et al. [50] discovered overexpression of *NFIB* promoted cell proliferation, migration and invasion, and inhibited cell apoptosis in gastric cancer cells through negatively regulating AKT/Stat3 axis. As our results demonstrated that NFIB, as a transcription factor, has the ability to regulate the *RIP2* activity, we further confirmed that *NFIB* participated in APEC-induced injuries through modulation of *RIP2* mediated signaling pathway in chicken HD11 cells. It has been demonstrated that deficiency of *RIP2* gene decreased NFκB activation and impaired expression of IL-6, TNF-α, and IP-10 in mice [60,61]. It is well known that the NFκB signaling pathway, a prototypical, pro-inflammatory signaling pathway, is generally important for the modulation of inflammatory or immune responses, cell survival, or cell proliferation [62]. Overexpression of *RIP2* gene could activate MAP kinases and augment caspase 8-mediated apoptosis [63,64,65], proving *RIP2* gene plays an important protective role in immune and inflammatory activities. Herein, we found that *NFIB* has the ability to regulate the activity of NFκB and IκB, which was blocked by knockdown of *RIP2*. Current results indicate that *NFIB* can impact the APEC-induced cell apoptosis and levels of inflammatory cytokines through regulating the RIP2/NFκB axis. Meanwhile, it also illustrates from the other side that *NFIB* might be an efficient regulator to avoid the potential damaging consequences of *RIP2* action.

## 4. Materials and Methods

### 4.1. Cell Culture

Chicken HD11 cells were from an animal genetic and breeding lab at Yangzhou University. The steady *RIP2* knockdown chicken HD11 cells have been established in the lab. In brief, according to the sequence of chicken *RIP2* gene, three RNA interference target sequences and one negative control sequence were designed for the *RIP2* gene, recombined with the pLVshRNA-EGFP(2A) Puro interference vector, and then transiently transfected into chicken HD11 cells. Interference efficiency of each target on *RIP2* was tested by RT-qPCR, and the recombinant vector with high interference efficiency was packaged with lentivirus to transfect chicken HD11 cells. The transfected HD11 cells were passaged for several generations. Then, RT-qPCR and Western blot were used to detect the expression changes of *RIP2*. Chicken HD11 cells were maintained in Dulbecco’s modified eagle medium (DMEM) (Thermo Fisher Scientific, Inc., Waltham, MA, USA), supplemented with 10% fetal bovine serum (FBS) (Gibco, Grand Island, NY, USA) and 1% penicillin streptomycin (Gibco, Grand Island, NY, USA). Cells were cultured in a humidified environment with 5% CO_2_ and 95% air at 37 °C. DF1 cells were purchased from ATCC. The culture condition of DF1 cells was the same with HD11 cells. For infection, cells were challenged with 0.1 mL containing 1 × 10^8^ colony forming units (CFUs) of APEC O78 for 24 h.

### 4.2. RT-qPCR

Total RNA was isolated from cells using Trizol reagent (Invitrogen, Carlsbad, CA, USA) on the basis of manufacturer’s instructions. Then, the RNA was reverse transcribed into cDNA using a Reverse Transcription Kit (Takara, Dalian, China). The One Step SYBR^®^ PrimeScript^®^ PLUS RTRNA PCR Kit (Takara, Dalian, China) was used for cDNA synthesis. RT-qPCR was conducted using a SYBR^®^ Premix Ex Taq^TM^ II Kit (Takara, Dalian, China) to evaluate the expression level of *RIP2*, *NFIB*, *IL1β*, *IL8*, *IL6*, *RELA* (*NF-κB* p65), and *IκBα*. Primer sequences were displayed in Table S2. RT-qPCR thermal cycling conditions were as follows: denaturation for 3 min at 95 °C, 40 cycles for 10 s at 95 °C, 58 °C for 30 s, and then 72 °C for 30 s. Relative expression of the above genes were calculated using the 2^−ΔΔCt^ method and *GAPDH* was utilized as an internal control.

### 4.3. Apoptosis Assay

Cell apoptosis was evaluated using an annexin V-PE/7-AAD apoptosis detection Kit (Vazyme, Nanjing, China). In brief, cells (5 × 10^5^ cells/well) were divided into different groups and seeded in 6-well plates. After transfection, the cells were placed in 500 µL of a binding buffer (Biosea Biotechnology, Beijing, China), treated using 5 µL of annexin V-PE and 10 µL of 7-AAD, maintained for 30 min at 25 °C in the dark. Stained cells were detected and analyzed using flow cytometry (Becton Dickinson, Franklin Lakes, NJ, USA). Each experiment was performed in triplicate.

### 4.4. Cell Viability Assay

Cell Counting Kit-8 (CCK-8) (Vazyme, Nanjing, China) was utilized to determine the viability of both the treated and untreated chicken HD11 cells. Transfected cells were placed in three replicates at a density of 1 × 10^5^ cells per well in a 96-well plate with 100 µL of medium and incubated for 48 h after treatment with APEC. Then, the cells were incubated for 2 h in 10 µL of CCK-8 solution. Absorbance (optical density, OD) was assessed at 450 nm using a microplate reader (DR-200Bs, Diatek, Wuxi, China).

### 4.5. Cell Transfection and Dual-Luciferase Reporter Assay for RIP2 Promoter

When the cells reached 70–80% confluence, cells were incubated in 24-well plates. Cells were transfected with Lipofectamine™ 2000 reagent (Invitrogen, Carlsbad, CA, USA). To determine the core promoter of the *RIP2* gene, a series of promoter fragments were amplified through 5′ unidirectional deletion specific primers containing *Hind* III and *Xho* I restriction enzyme sites, respectively (Table S3). The PCR products were cloned into pGL3-basic luciferase reporter vector (Progema, Madison, WI, USA) using T4 DNA Ligase (Takara, Dalian, China). After enzyme digestion and sequencing identification, the recombinant plasmids were extracted using EndoFree Mini Plasmid Kit II (Tiangen, Beijing, China), and named pGL3-RIP2-P1 (−439/+38), pGL3-RIP2-P2 (−962/+38), pGL3-RIP2-P3 (−1389/+38), pGL3-RIP2-P4 (−1839/+38), pGL3-RIP2-P5 (−2300/+38), pGL3-RIP2-P6 (−2750/+38), and pGL3-RIP2-P7 (−3162/+38), respectively. To verify the promoter activity of different fragments, each recombinant plasmid (800 ng) was co-transfection with internal vector pRL-TK (20 ng) using Lipofectamine™ 2000 reagent according to the manufacturer’s protocol. After 48 h post-transfection, the luciferase activity was detected using the dual luciferase reporter assay system (Promega, Madison, WI, USA) and the pGL3-basic vector was used as a negative control.

### 4.6. Bioinformatics Prediction

To predict the *RIP2* promoter region, we used the database UCSC Genome Browser (http://genome.ucsc.edu/; 20 May 2021), NCBI (http://www.ncbi.nlm.nih.gov/gene/; 20 May 2021), Promoter 2.0 (https://services.healthtech.dtu.dk/service.php?Promoter-2.0; 20 May 2021) and Promoter Scan (https://www-bimas.cit.nih.gov/molbio/proscan; 20 May 2021). The database of PROMO (http://alggen.lsi.upc.es/cgi-bin/promo_v3/promo/promoinit.cgi?dirDB=TF_8.3; 20 August 2021) and AliBaba 2.1 (http://gene-regulation.com/pub/programs/alibaba2/index.html; 20 August 2021) were used to predict the transcription factor binding sites within *RIP2* promoter.

### 4.7. Construction of Plasmids

We used GenePharma (Shanghai, China) to synthesize the following plasmids: *RIP2*, *NFIB,* and *GATA1* full length (pcDNA3.1-RIP2, pcDNA3.1-NFIB, and pcDNA3.1-GATA1) plasmid; siRNA *NFIB* (Si-NFIB-1, -2, and -3) plasmids (Table S4); and small hairpin *RIP2* (Sh-RIP2) plasmid (Table S5). The Lipofectamine™ 2000 reagent (Invitrogen, Carlsbad, CA, USA) was used for the cell transfection according to the manufacturer’s instructions. After chicken HD11 cells were transfected with pcDNA3.1-NFIB for 72 h, or with Si-NFIB or Sh-RIP2 for 48 h, differently treated cells were challenged with or without 0.1 mL containing 1 × 10^8^ colony forming units (CFUs) of APEC O78 for 24 h, and collected for further study. For experiments with co-transfection of pcDNA3.1-NFIB and Sh-RIP2, cells were first transfected with pcDNA3.1-NFIB for 24 h and then transfected with Sh-RIP2. After 48 h, cells were collected. Each experiment was performed at least three times.

### 4.8. CHIP-PCR

To determine whether transcription factor NFIB can bind to the *RIP2* promoter, a CHIP-PCR assay Kit (Millipore, MA, USA) was used to perform experiment. Briefly, cells (5~20 × 10^7^) were cross-linked using 1% formaldehyde, followed by scraping into ice-cold PBS with protease inhibitors. Next, the cells were collected and resuspended in lysis buffer (20 mM HEPES, pH = 7.9, 420 mM NaCl, 0.2 mM EDTA, 0.5% NP-40, 25% glycerol, 1.5 mM MgCl_2_), followed by several brief periods of sonication with Ultrasonic Homogenizer (JY92-IIN, Scientz, Ningbo, China). One-third of the cell extract was kept as input sample and two-thirds of the cell extract was used as substrate for immunoprecipitation with anti-NFIB antibody (LS-B13531, IHC-plus). Reverse cross-linking was added with 5 M NaCl, after which the eluted DNA was extracted for PCR analysis. The primer sequences for the *RIP2* promoter were listed in Table S6.

### 4.9. Western Blot Analysis

Cells grown in 24-well plates were lysed on ice using 200 μL RIPA buffer (Beyotime Biotechnology, Shanghai, China) for 30 min. Next, the lysis mixtures were centrifuged and the supernatants were collected. BCA™ Protein Assay Kit (Pierce, Appleton, WI, USA) was used for quantification of proteins. Then, proteins were subjected to sodium dodecyl sulfate-polyacrylamide gel (SDS-PAGE) and electrophoretically transferred to PVDF membranes. Afterwards, membranes were blocked in 5% bovine serum albumin (BSA) for 2 h at room temperature and then probed with primary antibodies at 4 °C overnight. The primary antibodies used in this study include anti-GAPDH (ab181602, Abcam, Cambridge, UK), anti-RIP2 (70R-10459, fitzgerald), anti-IL1β (abx132185, Abbexa, Cambridge, UK), anti-IL8 (abx100965, Abbexa), anti-IL6 (abx177189, Abbexa), anti-NFκB p65 (10745-1-AP, Proteintech, Wuhan, China), and anti-IκBα (10268-1-AP, Proteintech). The primary antibodies were used at a dilution of 1:1000. Then, the membranes were incubated with secondary antibodies marked by horseradish peroxidas (Sigma-Aldrich, St. Louis, MI, USA) at a 1:10,000 dilution at room temperature for 2 h. Immunoblots were visualized by enhanced chemiluminescence (ECL kit, Santa Cruz Biotechnology, Dallas, TX, USA). The images were analyzed using Image Lab™ Software (Bio-Rad, Hercules, CA, USA).

### 4.10. Nitric Oxide Production Assay

Chicken HD11 cells were incubated for 24 h post treatment (Control, APEC, pcDNA3.1-NFIB, Sh-RIP2, pcDNA3.1-NFIB+APEC, and APEC+pcDNA3.1-NFIB+Sh-RIP2), then NO production in the cell supernatant was determined using the Griess reagent Kit (Molecular Probes, Carlsbad, CA, USA). Supernatant was mixed with Griess reagents and incubated for 30 min at room temperature in dark conditions, and then measured at 540 nm on a Microplate Reader (DR-200Bs, Diatek). The absorbance values were compared to the sodium nitrite standard curve to determine nitrite concentrations (µM).

### 4.11. Statistical Analysis

All experiments were repeated three or five times. The results of multiple experiments are presented as the mean ± SD. Statistical analyses were performed using JMP 15.2.1 software [66]. Diagrams were generated in Graphpad Prism 6.0 statistical software (GraphPad Software, San Diego, CA, USA). Statistical analysis was carried out using a one-way analysis of variance (ANOVA) followed by least significant difference (LSD) test. A *p*-value of <0.05 was considered for a statistically significant result.

## 5. Conclusions

In conclusion, we identified knockdown of *RIP2* significantly improved cell viability and suppressed the apoptosis of APEC-stimulated cells. Additionally, we analyzed and cloned 5′ promoter region of *RIP2*, discovered the core promoter region at −2300 bp/−1839 bp, and identified the transcription factor NFIB binding to this region to regulate *RIP2* gene expression. Moreover, it was found that *NFIB* was up-regulated in APEC-injured chicken HD11 cells, and overexpression of *NFIB* increased the cell apoptosis and inflammation induced by APEC exposure in chicken cells. However, knockdown of *RIP2* attenuated APEC-induced injuries in chicken cells. The underlying mechanism of the immunological inflammation inhibition role of *NFIB* might be through regulation of *RIP2*. Our findings might provide new clues for understanding the role of *NIFB* and *RIP2* in the progression of APEC infection, prevent excessive inflammation, and offer a potential new target for therapeutic approach to the APEC infection.

## Data Availability

The data will be available from the corresponding author upon request.

## Supplementary Materials

The following are available online at https://www.mdpi.com/article/10.3390/ijms23073814/s1.
