# Supplementary material for: Transcriptional Regulation of RIP2 Gene by NFIB Is Associated with Cellular Immune and Inflammatory Response to APEC Infection
Source: Int J Mol Sci. 2022 Mar 30;23(7):3814. doi: 10.3390/ijms23073814 (PMC8998712; doi:10.3390/ijms23073814)
Supplement: Supplementary file 1 [file ijms-23-03814-s001.zip › Table S1.pdf]

Table S1. The detailed information of the used datasets for selecting the common transcriptomic response upon APEC infection in different immune tissues

Accession number	Title	Platform
GSE67302	Avian pathogenic Escherichia coli (APEC) infection alters bone marrow transcriptome in chickens	GPL16133 Illumina HiSeq 2000 (Gallus gallus)
GSE69014	Thymus transcriptome reveals novel pathways in response to avian pathogenic Escherichia coli infection	GPL16133 Illumina HiSeq 2000 (Gallus gallus)
GSE70334	Novel Pathways Revealed in Bursa of Fabricius Transcriptome in Response to Extraintestinal Pathogenic Escherichia coli (ExPEC) Infection	GPL16133 Illumina HiSeq 2000 (Gallus gallus)
GSE31387	Transcriptome Response of Leukocytes from Chickens Infected with Avian Pathogenic Escherichia coli Identifies Pathways Associated with Resistance	GPL6413 Chicken 44K custom Agilent microarray (2nd version)
GSE25511	Spleen transcriptome response to infection with avian pathogenic Escherichia coli in broiler chickens	GPL6413 Chicken 44K custom Agilent microarray (2nd version)
