# Supplementary material for: Transcriptional Regulation of RIP2 Gene by NFIB Is Associated with Cellular Immune and Inflammatory Response to APEC Infection
Source: Int J Mol Sci. 2022 Mar 30;23(7):3814. doi: 10.3390/ijms23073814 (PMC8998712; doi:10.3390/ijms23073814)
Supplement: Supplementary file 1 [file ijms-23-03814-s001.zip › Table S2.pdf]

Table S2. Primers for RT-qPCR

Gene	Genbank accession No.		Sequence (5'-3')	Tm value	Product length (bp)
GAPDH	NM_204305.1	sense	GTCGGAGTCAACGGATTTGG	60.1	177
		antisense	GTTCTCAGCCTTGACAGTGCC	59.6	
RIP2	NM_001030943.1	sense	CTCGAACCAGTCCTGAGAACG	59.7	175
		antisense	AAGCGGATGTTTCCTCTTGG	59.3	
NFIB	NM_205272.2	sense	AGCATCACCACCCAGGAATC	59.3	207
		antisense	TGGACGGGTCGTAAGAAGGTA	59.7	
IL1β	NM_204524.1	sense	GTGGCCATGACCAAACTGCT	60.5	162
		antisense	GAAGGACTGTGAGCGGGTGT	59.6	
IL6	NM_204628.1	sense	GAGGAGAAATGCCTGACGAAG	59	187
		antisense	CCGAGTCTGGGATGACCACT	59.3	
IL8	NM_205018.1	sense	ATTCAAAATGTGAACCTCACCC	58.2	152
		antisense	TTGGTGTCTGCCTTGTCCAG	58.9	
p65	NM_205129.1	sense	CAGCCCATCTATGACAACCG	60.1	152
		antisense	TCAGCCCAGAAACGAACCTC	59.6	
IκBα	NM_001001472	sense	CACTTGGCCGTAGACCTTCA	58.6	145
		antisense	TCAGCTGCTCCTGTATGCTG	59.2	
