# Supplementary material for: Transcriptional Regulation of RIP2 Gene by NFIB Is Associated with Cellular Immune and Inflammatory Response to APEC Infection
Source: Int J Mol Sci. 2022 Mar 30;23(7):3814. doi: 10.3390/ijms23073814 (PMC8998712; doi:10.3390/ijms23073814)
Supplement: Supplementary file 1 [file ijms-23-03814-s001.zip › Table S3.pdf]

Table S3. Primers for deletion fragments of chicken *RIP2* gene promoter region

Name	5′ - 3′ primer sequence	Product length (bp)
pGL3-RIP2-P1-F	CGTGCTAGCCCGGG<u>CTCGAG</u>TGAAATGAGCAAATGCAGCCG	440
pGL3-RIP2-P1-R	AACAGTACCGGAATGCC<u>AAGCTT</u>CCGGCAGCTTGCTAGAGGG	
pGL3-RIP2-P2-F	CGTGCTAGCCCGGG<u>CTCGAG</u>TTGTGCCTTCAGTCGCATGCC	927
pGL3-RIP2-P2-R	AACAGTACCGGAATGCC<u>AAGCTT</u>CCGGCAGCTTGCTAGAGGG	
pGL3-RIP2-P3-F	CGTGCTAGCCCGGG<u>CTCGAG</u>GAACAGGCTGCCCAGAGAAGC	1390
pGL3-RIP2-P3-R	AACAGTACCGGAATGCC<u>AAGCTT</u>CCGGCAGCTTGCTAGAGGG	
pGL3-RIP2-P4-F	CGTGCTAGCCCGGG<u>CTCGAG</u>ACAGTGCTACTGTCAGGAGTC	1840
pGL3-RIP2-P4-R	AACAGTACCGGAATGCC<u>AAGCTT</u>CCGGCAGCTTGCTAGAGGG	
pGL3-RIP2-P5-F	CGTGCTAGCCCGGG<u>CTCGAG</u>GGGGCACTGGAGATTCATGAT	2301
pGL3-RIP2-P5-R	AACAGTACCGGAATGCC<u>AAGCTT</u>CCGGCAGCTTGCTAGAGGG	
pGL3-RIP2-P6-F	CGTGCTAGCCCGGG<u>CTCGAG</u>GGTCTGGAGCCCCCAGTACAA	2751
pGL3-RIP2-P6-R	AACAGTACCGGAATGCC<u>AAGCTT</u>CCGGCAGCTTGCTAGAGGG	
pGL3-RIP2-P7-F	CGTGCTAGCCCGGG<u>CTCGAG</u>TGATAAACCACTGAAACTAA	3201
pGL3-RIP2-P7-R	AACAGTACCGGAATGCC<u>AAGCTT</u>CCGGCAGCTTGCTAGAGGG	

The underline is enzyme digestion site. *Xho*I recognize sequence CTCGAG; *Hind*III recognize sequence AAGCT
