# Supplementary material for: Transcriptional Regulation of RIP2 Gene by NFIB Is Associated with Cellular Immune and Inflammatory Response to APEC Infection
Source: Int J Mol Sci. 2022 Mar 30;23(7):3814. doi: 10.3390/ijms23073814 (PMC8998712; doi:10.3390/ijms23073814)
Supplement: Supplementary file 1 [file ijms-23-03814-s001.zip › Table S4.pdf]

Table S4. siRNA information for NFIB

Name	Target sequence
Si-NFIB-1	GGAGCAAGATTCTGGACAA
Si-NFIB-2	GCACAGAGTGTCATCTCAA
Si-NFIB-3	CCCAAGGAACTGGAGTGAA
