# Supplementary material for: Transcriptional Regulation of RIP2 Gene by NFIB Is Associated with Cellular Immune and Inflammatory Response to APEC Infection
Source: Int J Mol Sci. 2022 Mar 30;23(7):3814. doi: 10.3390/ijms23073814 (PMC8998712; doi:10.3390/ijms23073814)
Supplement: Supplementary file 1 [file ijms-23-03814-s001.zip › Table S5.pdf]

Table S5. shRNA information for *RIP2*

ID	5’	stem	loop	stem	3’
NC-F	GATCC	GACGAAAGTCAAGACTTCTATTCAA	TTCAAGAGA	TTGAATAGAAGTCTTGACTTTCGTC	TTTTTTG
NC-R	AATTCAAAAAA	GACGAAAGTCAAGACTTCTATTCAA	TCTCTTGAA	TTGAATAGAAGTCTTGACTTTCGTC	G
shRNA1-F	GATCC	ACAGTGATAGAAACCACCTTCTAAA	TTCAAGAGA	TTTAGAAGGTGGTTTCTATCACTGT	TTTTTTG
shRNA1-R	AATTCAAAAAA	ACAGTGATAGAAACCACCTTCTAAA	TCTCTTGAA	TTTAGAAGGTGGTTTCTATCACTGT	G
shRNA2-F	GATCC	GACTTAAGTGAAACCAGCTTATCAA	TTCAAGAGA	TTGATAAGCTGGTTTCACTTAAGTC	TTTTTTG
shRNA2-R	AATTCAAAAAA	GACTTAAGTGAAACCAGCTTATCAA	TCTCTTGAA	TTGATAAGCTGGTTTCACTTAAGTC	G
shRNA3-F	GATCC	ACAAACAGCTAGGACTTCAACCTTA	TTCAAGAGA	TAAGGTTGAAGTCCTAGCTGTTTGT	TTTTTTG
shRNA3-R	AATTCAAAAAA	ACAAACAGCTAGGACTTCAACCTTA	TCTCTTGAA	TAAGGTTGAAGTCCTAGCTGTTTGT	G
