# Supplementary material for: Transcriptional Regulation of RIP2 Gene by NFIB Is Associated with Cellular Immune and Inflammatory Response to APEC Infection
Source: Int J Mol Sci. 2022 Mar 30;23(7):3814. doi: 10.3390/ijms23073814 (PMC8998712; doi:10.3390/ijms23073814)
Supplement: Supplementary file 1 [file ijms-23-03814-s001.zip › Table S6.pdf]

Table S6. The primer for CHIP-PCR experiment

**Gene**		**Sequence（5`-3`）**	**Tm value**	**CG%**	**Product length (bp)**
RIP2	sense	TTCTGTGATGAGGCTGTGAACC	59.9	50	196
	antisense	GTGATGTGGCATAAGCTGCAG	59.1	52.4	
